# M3-RGB: An Imaging Sensor System Using Multicore, Multimode Optical Fiber and Neural Networks

**DOI:** 10.3390/s26175582

**Published:** 2026-09-02

**Authors:** Seigo Ito, Isamu Takai, Akari Kawasaki, Tadashi Ichikawa, Shin Motooka, Minoru Tanaka

**Affiliations:** Toyota Central R&D Labs., Inc., 41-1, Yokomichi, Nagakute 480-1192, Aichi, Japan; takai@mosk.tytlabs.co.jp (I.T.); akari@mosk.tytlabs.co.jp (A.K.); t-ichikawa@mosk.tytlabs.co.jp (T.I.); shin.motooka.hn@mosk.tytlabs.co.jp (S.M.); m-tanaka@mosk.tytlabs.co.jp (M.T.)

**Keywords:** multicore multimode optical fiber, incoherent-light fiber imaging, passive optical image relay, fiber-based imaging, image reconstruction, computational imaging, deep learning, vision transformer

## Abstract

Conventional image acquisition requires an electrically powered image sensor to be placed directly behind the camera lens, constraining camera placement. To overcome this issue, we introduce M3-RGB as an incoherent-light fiber imaging system in which a multicore, multimode optical fiber passively relays lens images to a remotely located image sensor. Unlike conventional approaches, M3-RGB is designed to operate directly on incoherent light and requires no electrical power or active components at the sensing interface. Because propagation through the fiber yields spatially scrambled patterns, a neural network is used to reconstruct the original scene by exploiting the spatial locality preserved by the multicore structure. In a controlled optical bench setup, where a liquid crystal display monitor displays road-scene images, we construct a paired dataset of scrambled and ground-truth images and quantitatively evaluate reconstruction performance across different fiber core counts, fiber lengths, and calibration settings, utilizing the peak signal-to-noise ratio and structural similarity index measure as performance metrics. By decoupling imaging electronics from the sensing point, this passive remote image relay approach may expand sensor placement options for potential applications such as all-around perception for mobile robots and autonomous vehicles, surveillance, and inspection in confined spaces. Evaluations in real outdoor environments constitute future work.

## 1. Introduction

Image acquisition conventionally requires an electrically powered image sensor to be positioned directly behind the lens, indicating that imaging electronics must be co-located with the optical aperture. This type of tight coupling constrains camera placement as every sensing point requires power and data cabling, must dissipate heat, and must reserve physical space for the lens-and-sensor assembly. A passive optical relay that transports lens images to a remotely located image sensor without any electronics or electrical power at the sensing interface would substantially relax these constraints. In this study, we investigate incoherent-light fiber imaging through a multicore, multimode optical fiber, where the fiber passively relays the scenes captured by the lens to a remote image sensor for computational reconstruction.

One important application domain motivating this study is multicamera perception for autonomous mobile robots and self-driving vehicles. These types of systems are becoming increasingly reliant on large numbers of cameras to achieve safe and comprehensive perception, increasing hardware costs and complicating power and data cabling, thermal management, and the physical placement of lenses and sensors on platforms. For example, the AutoVision project [1] employs 16 surround-view cameras, including red–green–blue (RGB) and near-infrared sensors, to achieve robust vehicle localization and dense 3D scene understanding, while the nuScenes dataset [2] relies on six synchronized surround-view cameras to provide full 360-degree coverage. These examples illustrate how the number and placement of image sensors directly drive system cost and integration complexity, motivating the development of sensing architectures that decouple imaging electronics from the optical sensing point.

**Figure 1:**
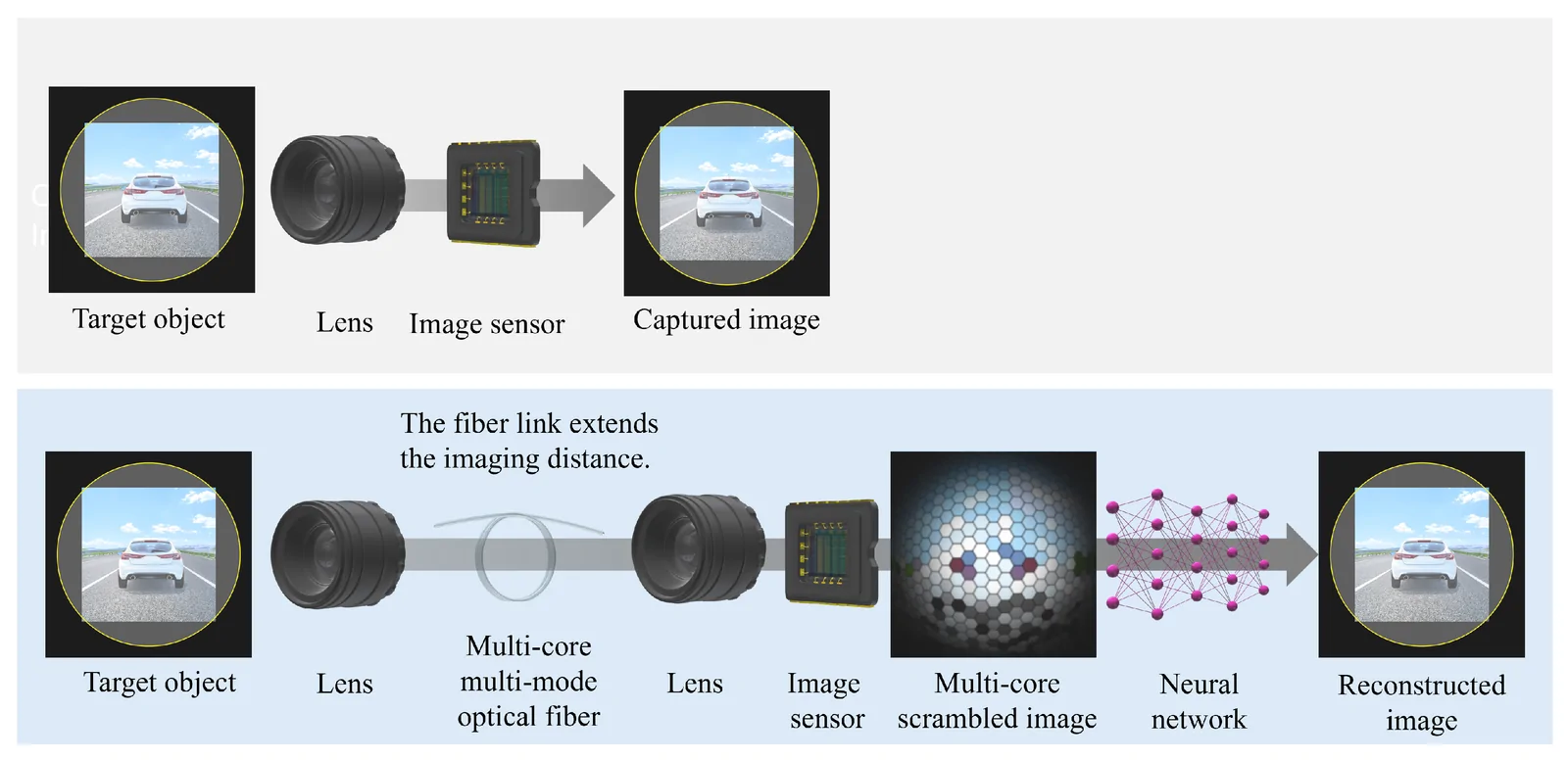
(**Upper**) Conventional camera system. (**Lower**) Overview of the M3-RGB architecture. The M3-RGB system employs multicore, multimode optical fibers, enabling image transmission with an extended distance between the lens and image sensor, without electrical power along the fiber link.

Accordingly, we propose an imaging system called M3-RGB, where “M3” is derived from the “multicore, multimode” features of the optical fiber that is at the heart of our system. Unlike a conventional camera, M3-RGB does not project scene light directly onto the image sensor through a lens. Instead, the system first transfers the light captured by the lens into a multicore, multimode optical fiber, transmits it over a distance, and then projects it onto a remotely located image sensor (see Figure 1). In this context, the term “passive” refers to the distal sensing interface and fiber link, which require no electrical power or active components. The image sensor itself resides remotely and receives electrical power, as in a conventional camera. Furthermore, we designed M3-RGB to operate directly on incoherent light, without any laser source or spatial light modulator (SLM). Section 2 presents detailed comparisons with coherent, laser-based fiber imaging approaches.

**Figure 2:**
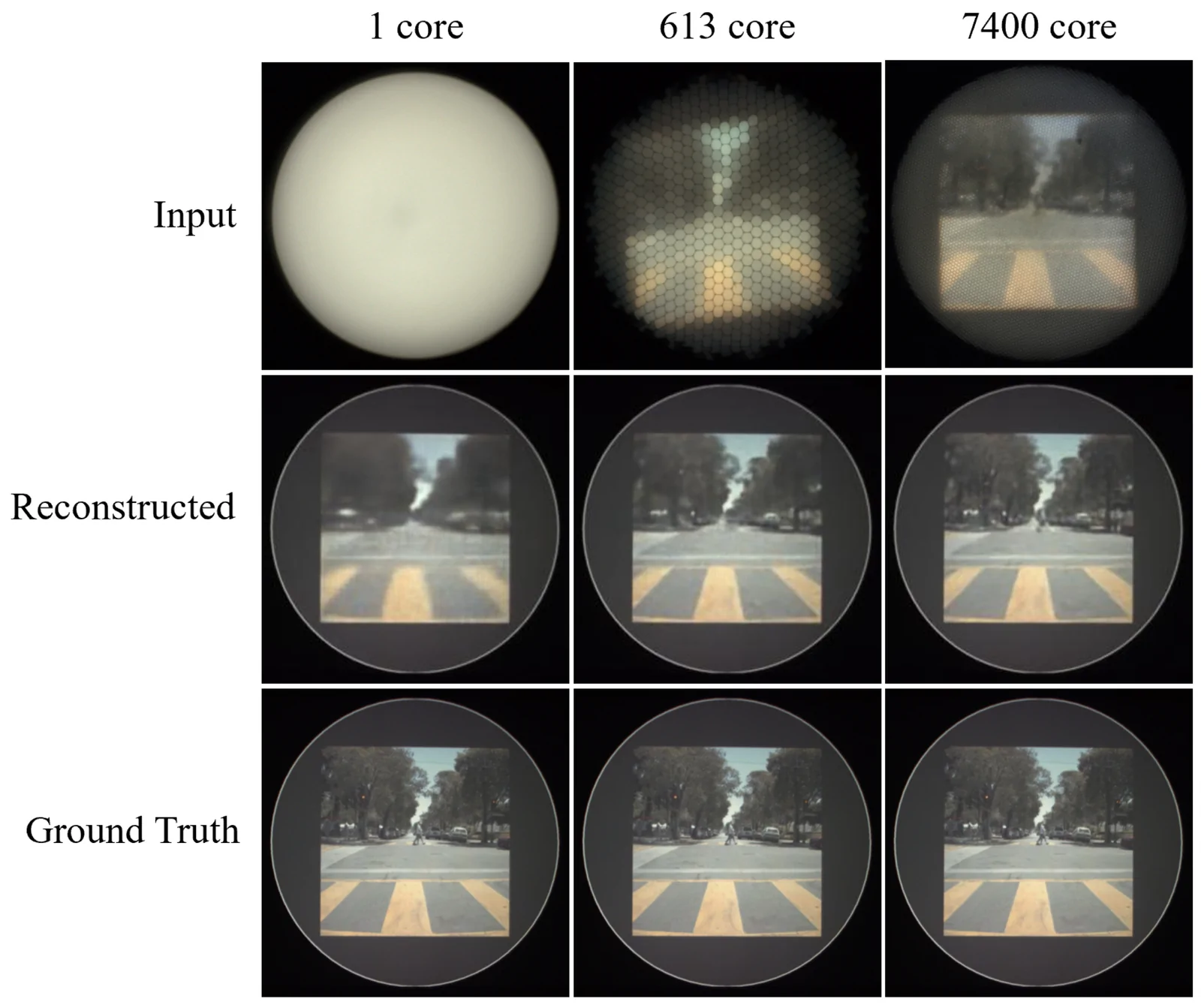
Examples of input, reconstructed, and ground-truth images obtained with the M3-RGB system. A larger number of fiber cores consistently enhances reconstruction fidelity.

The image that emerges after multicore, multimode fiber transmission appears as a scrambled pattern (as the image obtained under incoherent scene light), wherein each object point excites many guided modes, whose intensity contributions are added in the output. In our configuration, we interpret this distortion as intermodal dispersion and mode mixing, which broadens the point response within each core, while the discrete core structure segments incoming images across cores [3,4] (see Figure 2). Despite the presence of this type of distortion, the scrambled images retain partial information from the original optical signals. M3-RGB reconstructs original scenes from this optical channel using a neural network.

The main contributions of this study can be summarized as follows:

- We propose M3-RGB as an incoherent-light, passive remote image-relay system based on a multicore, multimode optical fiber that requires neither an auxiliary laser source nor electrical power at the sensing interface.
- We construct a paired dataset of scrambled and ground-truth road-scene images, enabling supervised image reconstruction through the optical channel of a multicore, multimode fiber.
- We quantitatively evaluate image reconstruction performance at different fiber core counts, fiber lengths, and calibration settings.

This study focuses on controlled optical bench characterization using liquid crystal display (LCD) images of the road-scene and condition-specific reconstruction models. Outdoor dynamic scenes and cross-configuration generalization constitute future work.

The remainder of this study is organized as follows. Section 2 reviews related studies on multicamera perception systems and fiber-based imaging. Section 3 describes the M3-RGB sensor system. In Section 4, the proposed method is evaluated based on quantitative experiments. Section 5 discusses the scope and limitations of this study. Section 6 presents envisioned application scenarios and directions for future research. Finally, Section 7 concludes the study.

## 2. Related Work

### 2.1. Multicamera Perception Systems

As a motivating application domain, autonomous mobile robots and self-driving vehicles have become increasingly reliant on multiple image sensors for omnidirectional perception [5,6,7], amplifying the costs and integration constraints discussed in the previous section. By relaying lens images to a remotely located image sensor passively, M3-RGB decouples imaging electronics from the optical sensing point, thereby relaxing integration constraints, such as sensor placement and cable routing. However, we emphasize that this type of multicamera perception is merely one representative application scenario rather than the main focus of this study, the central contribution of which is incoherent-light image transmission and reconstruction through a multicore, multimode optical fiber.

### 2.2. Fiber-Based Imaging

A number of previous studies have investigated imaging systems utilizing multimode fibers, fiber bundles, and disordered fibers. For example, Caramazza et al. [3] presented a statistical approach for the high-quality imaging of arbitrary natural scenes transmitted through a multimode fiber. Using 50,000 ImageNet images, their model learns a single complex-valued inverse transmission matrix that maps output speckle patterns back to input images. This approach enables full-color, high-resolution video capture at 20 frames per second through fibers up to 10 m long, without requiring phase-sensitive measurements or deep neural networks. However, this method requires a continuous-wave laser, as an SLM modulates image information onto the laser beam, and the setup then transfers the modulated light into a multimode optical fiber. Similarly, the configuration proposed by Liu et al. [8] also requires a laser source in conjunction with a multimode fiber. Furthermore, the imaging principle of their method differs fundamentally from ours, as the intermodal dispersion of a kilometer-long fiber driven by a pulsed laser converts spatial information into 1D temporal waveforms and recovers images from these waveforms rather than from spatial intensity patterns captured by an image sensor. In contrast, our M3-RGB system does not require any laser source and operates solely based on incoherent scene light, directly capturing spatial intensity patterns at the fiber’s output.

Zheng et al. [9] introduced a neural-network-based method for the single-ended recovery of multimode optical fiber transmission matrices (TMs) using multiwavelength reflection-mode measurements, thereby eliminating the need for distal-end access. Their findings advance ultrathin, high-speed endoscopic and remote imaging by enabling real-time TM estimation from a single fiber end. However, the goal of their study was to calibrate the fiber channel itself to recover its transmission matrix as a precursor to imaging, whereas M3-RGB was intended to perform end-to-end image reconstruction directly from captured fiber outputs, without estimating a transmission matrix.

Previous studies have also applied deep learning to image reconstruction based on other fiber-optic media. Hu et al. [10] demonstrated the unsupervised full-color image reconstruction of cellular specimens transmitted through a disordered optical fiber, successfully recovering high-fidelity images without paired training data. Shao et al. [11] enhanced the resolution of fiber bundle (coherent multicore bundle) imaging using a deep network that suppresses the honeycomb pixelation artifacts inherent to fiber bundles. Both methods target microscopy and endoscopy, where a dedicated, controlled light source illuminates the target. For example, active illumination lighted the specimen in both the transmission and reflection imaging modes as demonstrated in [10], while a dedicated white light-emitting diode source illuminated the target in [11]. Consequently, the captured signals depend on this purpose-built illumination rather than on the light emanating from the scene naturally. In contrast, M3-RGB reconstructs road-scene image content acquired under controlled conditions, forming images from incoherent light from LCD-displayed road-scene images in the experiments presented herein and, in principle, from actual scenes without requiring laser illumination, an SLM, or a dedicated illumination unit at the fiber’s sensing interface. This distinction in illumination requirements, in combination with our use of a multicore, multimode fiber, distinguishes our setting from previous illumination-dependent fiber imaging approaches.

Closely related to our setting, Fukushima et al. [4] proposed a streamlined direct image-transmission system. Similar to M3-RGB, their system relies only on lenses, an optical fiber, an image sensor, and a reconstruction network, and operates on incoherent scene light without a laser source. However, their system uses a *single-core* (plastic) optical fiber, and focus is placed on the reconstruction of relatively simple content, such as handwritten characters and digits, as well as evaluations of robustness to fiber bending, twisting, and temperature variation. In contrast, M3-RGB employs a *multicore*, multimode optical fiber and reconstructs general road-scene image content, which is a reconstruction problem with higher spatial complexity and natural image variability in terms of texture, color, and fine spatial detail. In summary, Fukushima et al. demonstrated direct ambient-light image transmission through a single-core plastic fiber and its environmental robustness, whereas our focus lies on a multicore, multimode fiber and the characterization of core count and length dependence for natural road-scene image reconstruction. These differences in fiber structure (single-core versus multicore), target content (simple characters versus general scenes), and evaluation axis (feasibility and robustness demonstration versus systematic core count and length characterization) distinguish our study from this closely related study.

Table 1 summarizes the representative fiber-based imaging studies reviewed above and the relative position of M3-RGB. The proposed method differs from these studies in the following respects. First, unlike coherent approaches [3,8] and illumination-dependent approaches [9,10,11], M3-RGB operates solely on incoherent scene light, requiring neither a laser source, an SLM, nor dedicated illumination at the sensing interface. Second, while previous studies have demonstrated incoherent light transmission only with a single-core fiber and simple content [4], M3-RGB employs a multicore, multimode fiber and reconstructs natural road-scene images. Third, in terms of evaluation axes, M3-RGB provides a systematic characterization of reconstruction performance across different core counts, fiber lengths, and calibration settings, which have not been addressed by any of the reviewed studies. Rather than claiming novelty based on the use of road-scene imagery alone, we define the contribution of this study as the combination of these aspects. To the best of our knowledge, no previous study on fiber-based image reconstruction has reported the combination of a passive incoherent display/scene light, multicore, multimode plastic optical fibers (POFs), natural road-scene content, and systematic core-count/length/calibration characterization. We consider road-scene content as one of the evaluation targets within this combination, rather than the primary contribution of our study.

## 3. M3-RGB

In this section, we introduce the proposed M3-RGB sensor system. We first present an overview of the system and the physical origin of the scrambled measurements captured by a multicore, multimode optical fiber and then present the reconstruction network architecture and evaluate its training loss.

### 3.1. Overview

**Figure 3:**
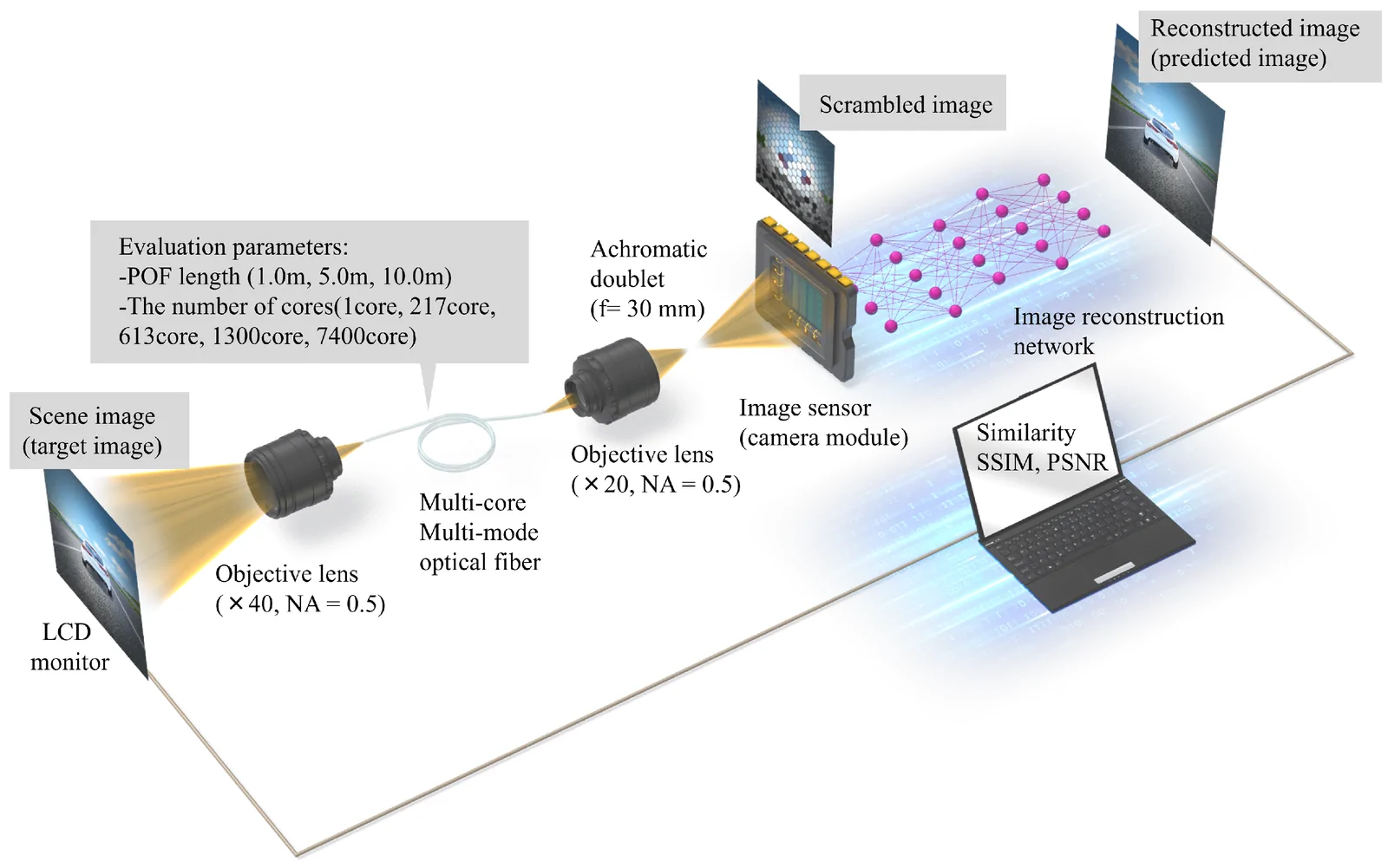
System overview of M3-RGB, illustrating the complete sensing pipeline, including the optical configuration.

This subsection presents an overview of the M3-RGB system (see Figure 1 and Figure 3). In conventional camera systems, the image sensor directly digitizes scene light passing through the lens. In contrast, M3-RGB first transfers the scene light transmitted through the lens into a multicore, multimode optical fiber to extend the distance between the lens and image sensor. After the light propagates through the fiber, the image sensor digitizes the received light. This setup allows M3-RGB to capture light transmitted through a single fiber, as well as (in principle) light from multiple fibers, utilizing a single image sensor, although this study evaluates only a single fiber end face. Section 6 discusses the multifiber configuration as a topic for future work. Previous studies have primarily relied on single-core, multimode optical fibers. To improve image reconstruction performance, in this study, we employ multicore, multimode optical fibers in addition to single-core fibers.

**Figure 4:**
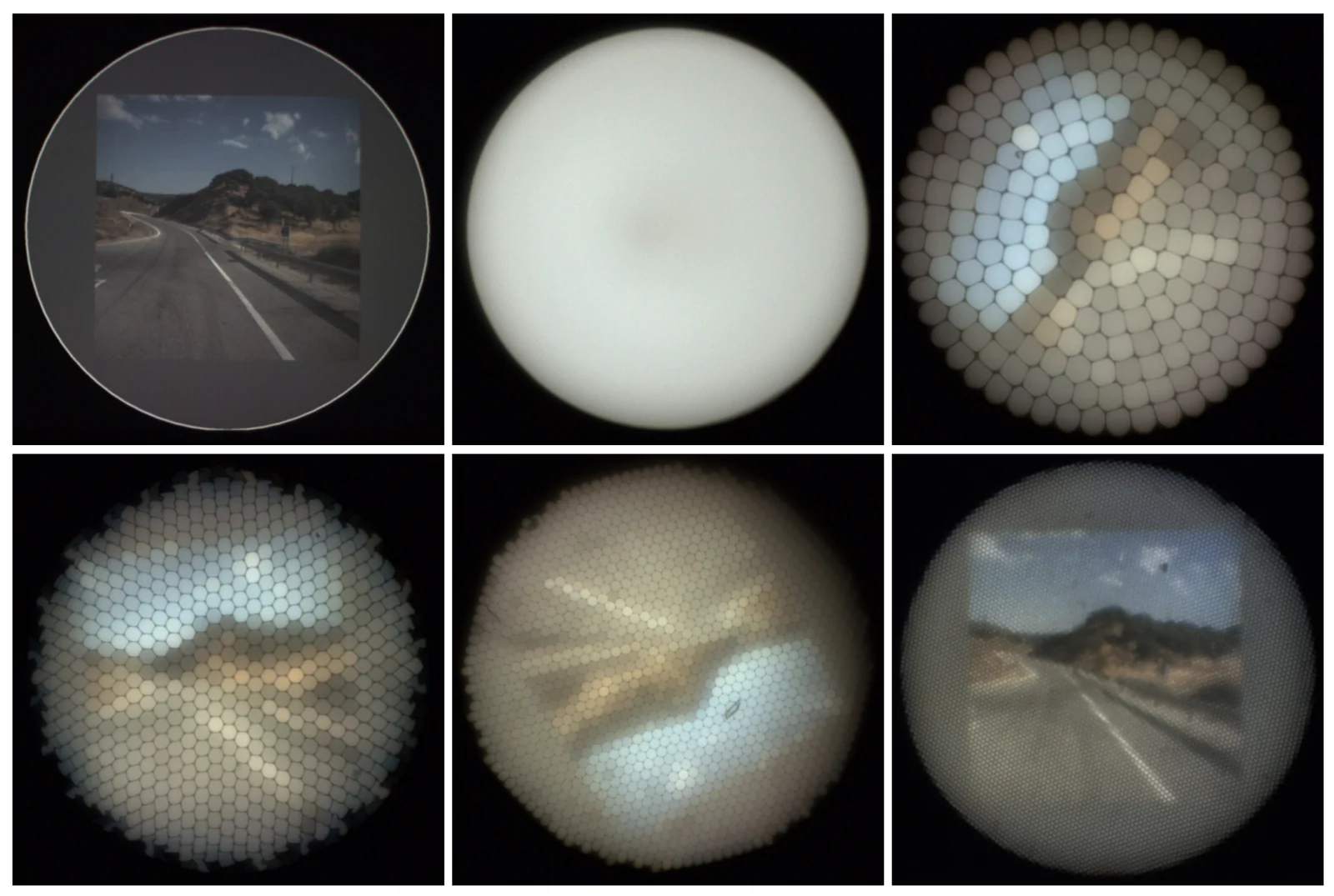
Example of a ground-truth image (**upper left**) and its scrambled counterparts captured through fibers with varying core counts: 1 core (**upper center**), 217 cores (**upper right**), 613 cores (**lower left**), 1300 cores (**lower center**), and 7400 cores (**lower right**).

Images captured by the sensor appear as scrambled patterns, as illustrated in Figure 4. This scrambling is a result of multiple optical effects within the fiber, that is, owing to the excitation of many guided modes at the entrance face, followed by intermodal dispersion and mode coupling during propagation. In a single-core fiber, the transmitted content is virtually indiscernible to the human eye. However, as the number of cores increases from 217 to 7400, the transmitted scene gradually becomes recognizable to the human eye (see Figure 4) because the scrambling effect remains spatially confined to the region corresponding to each individual core.

**Figure 5:**
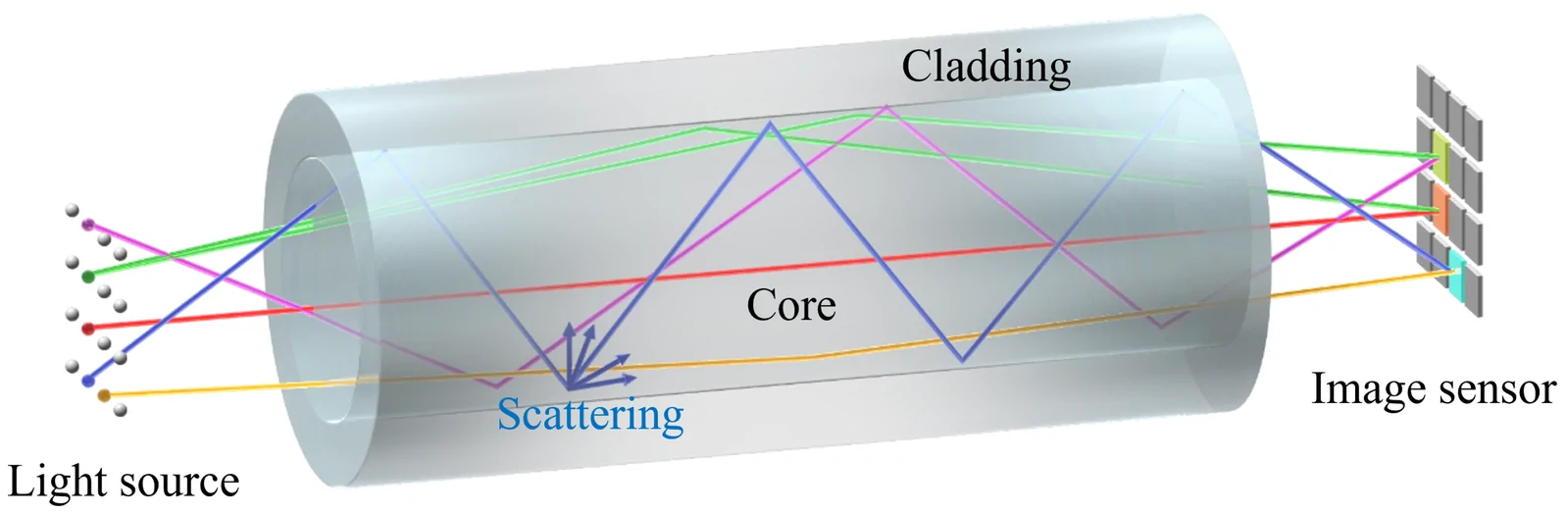
Conceptual illustration of incoherent light propagation in a single-core, multimode fiber. Light entering at different angles excites multiple guided modes; because the light is incoherent, these modes superimpose their intensity values, rather than forming a stable coherent speckle pattern under broadband illumination conditions. Intermodal dispersion in combination with mode coupling spreads each object point over a broad output distribution, yielding a scrambled intensity pattern at the sensor. Multicore, multimode fibers embed several isolated cores within a shared cladding, enabling parallel multimode propagation while segmenting images across cores.

Figure 5 presents a conceptual illustration of incoherent light propagation in a multimode optical fiber. Scene light from different sources enters the fiber at varying angles and positions, exciting multiple guided modes that travel along distinct paths and undergo repeated internal reflections within the fiber core. Because the incident scene light is incoherent, these excited modes do not form a stable coherent speckle pattern under the target broadband display illumination and camera exposure conditions. Instead, their intensity distributions are superimposed at the output. Therefore, we can interpret each object point as spreading over a broad output intensity distribution through intermodal (modal) dispersion, and the mode coupling induced by fiber bending and imperfections further enhances this spreading [3,4], strongly blurring the mapping between input positions and output intensities. In a multicore fiber, discrete cores sample and segment images, effectively dividing spatial information across cores while smearing it within each core. As a result, transmission partially degrades, instead of completely destroying, the original spatial structure. In the single-core case, this degradation is severe, whereas the multicore structure retains spatial locality at the core scale, and the output light forms a scrambled intensity pattern at the image sensor location. This process highlights the complexity of multimode fiber transmission and the challenge of image reconstruction.

By reconstructing scrambled images using the neural network architecture described in the following subsection, the proposed system can approximate the functionality of a conventional camera for image acquisition.

### 3.2. Architecture

This subsection presents the architecture of our neural network, which is designed to reconstruct original images from their scrambled counterparts. Previous studies on multimode optical-fiber-based imaging have employed architectures such as U-Net and fully convolutional networks (e.g., [9]). In this study, we adopt a multistage vision transformer (ViT)-based design as the reconstruction backbone. We employ an attention mechanism and multiple positional embeddings to model the correspondence between distorted optical patterns and the original spatial structure of the target scene.

**Figure 6:**
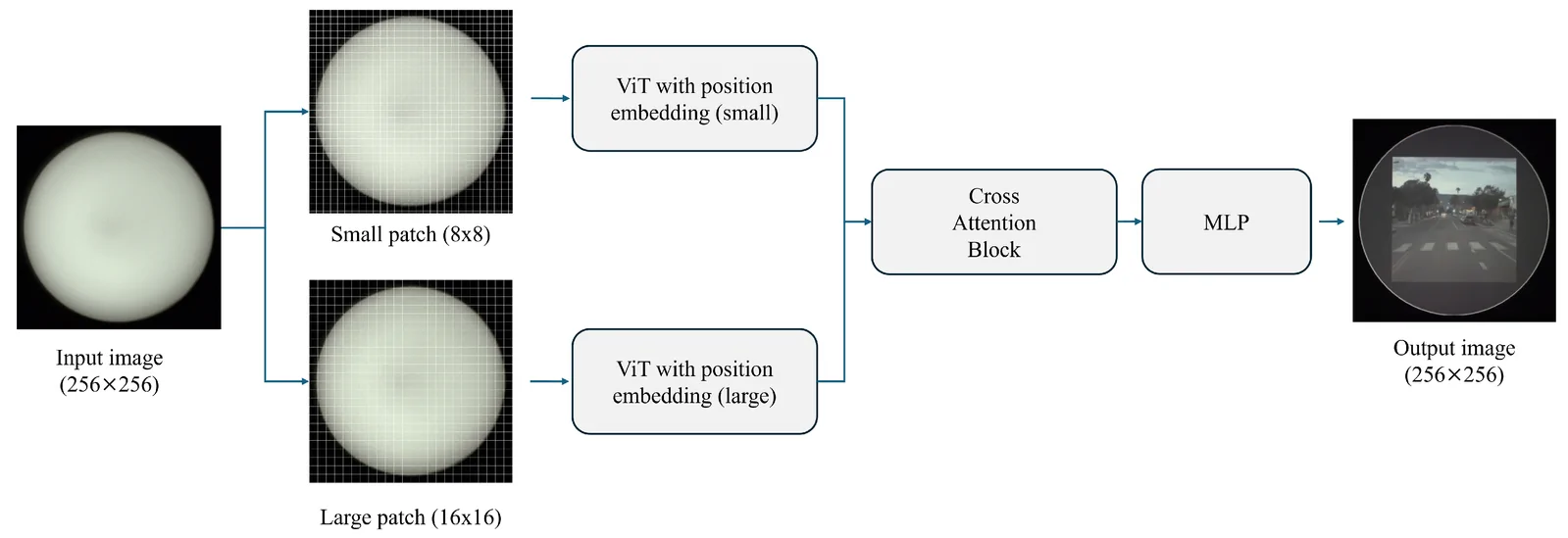
Network architecture of M3-RGB for image reconstruction using a ViT with different positional embeddings. The network tokenizes a scrambled image into both small and large patch sizes and processes it through separate ViT branches. An attention block fuses the outputs, and a multilayer perceptron refines them to reconstruct the original image.

Figure 6 illustrates the proposed reconstruction architecture, which is implemented in M3-RGB. M3-RGB tokenizes a scrambled input image at two different patch sizes and processes it using two parallel ViT branches: one branch that operates on small patches to extract fine-grained local details and another that operates on larger patches to capture broader contextual information. Each branch has its own positional embeddings. An attention-based fusion block followed by a multilayer perceptron then integrates the token representations from the two branches and maps the fused features back into the image domain to reconstruct the original scene. The training loss function is described below.

In M3-RGB, we employ a channel-weighted reconstruction loss function (Equations (1) and (2)) for image reconstruction through multimode fibers. This formulation incorporates channel-wise weights to account for wavelength-dependent propagation losses. We apply these weights to the per-pixel error term, allowing the model to emphasize channels that experience stronger attenuation during transmission. In Equation (2), *B* denotes the batch size, *C* denotes the number of color channels (here C=3), and *H* and *W* denote the image height and width, respectively. ${\hat{I}}_{b,c,i,j}$ and $I_{b,c,i,j}$ are the reconstructed and ground-truth pixel intensities, respectively, at channel *c* and spatial location (i,j) in the *b*th sample.

(1) $$s=(s_{\mathrm{red}},s_{\mathrm{green}},s_{\mathrm{blue}})$$

(2) $$L=\frac{1}{BCHW}\sum_{b=1}^{B}\sum_{c=1}^{C}\sum_{i=1}^{H}\sum_{j=1}^{W}s_{c}{\left({\hat{I}}_{b,c,i,j}-I_{b,c,i,j}\right)}^{2}$$

The weight vector s in Equation (1) consists of three weights corresponding to the color channels, and $s_{c}$ in Equation (2) denotes the weight for channel *c*. In practice, multimode fibers exhibit higher propagation loss in the R wavelength region, while G and B wavelengths experience comparatively lower losses. In all experiments, we used $s=\left(s_{\mathrm{red}},s_{\mathrm{green}},s_{\mathrm{blue}}\right)=\left(1.8,1.3,1.4\right)$ based on the propagation loss specification of the plastic multimode optical fiber, while accounting for the peak sensitivity wavelengths of the camera. Accordingly, the R channel, which experiences the strongest attenuation, receives the largest weight. We used the same weights for all fiber configurations, and this behavior was incorporated into our training process using the weighted formulation defined in Equation (2).

## 4. Experiments

To evaluate the proposed sensor system, we first constructed a paired dataset of ground-truth and scrambled road-scene images and then conducted three experiments to assess the effects of the following factors on reconstruction performance:

- Number of fiber cores;
- Fiber length;
- Rotation calibration.

The following subsections explain the dataset construction process and each of our experiments.

### 4.1. Dataset Construction and Experimental Setup

**Figure 7:**
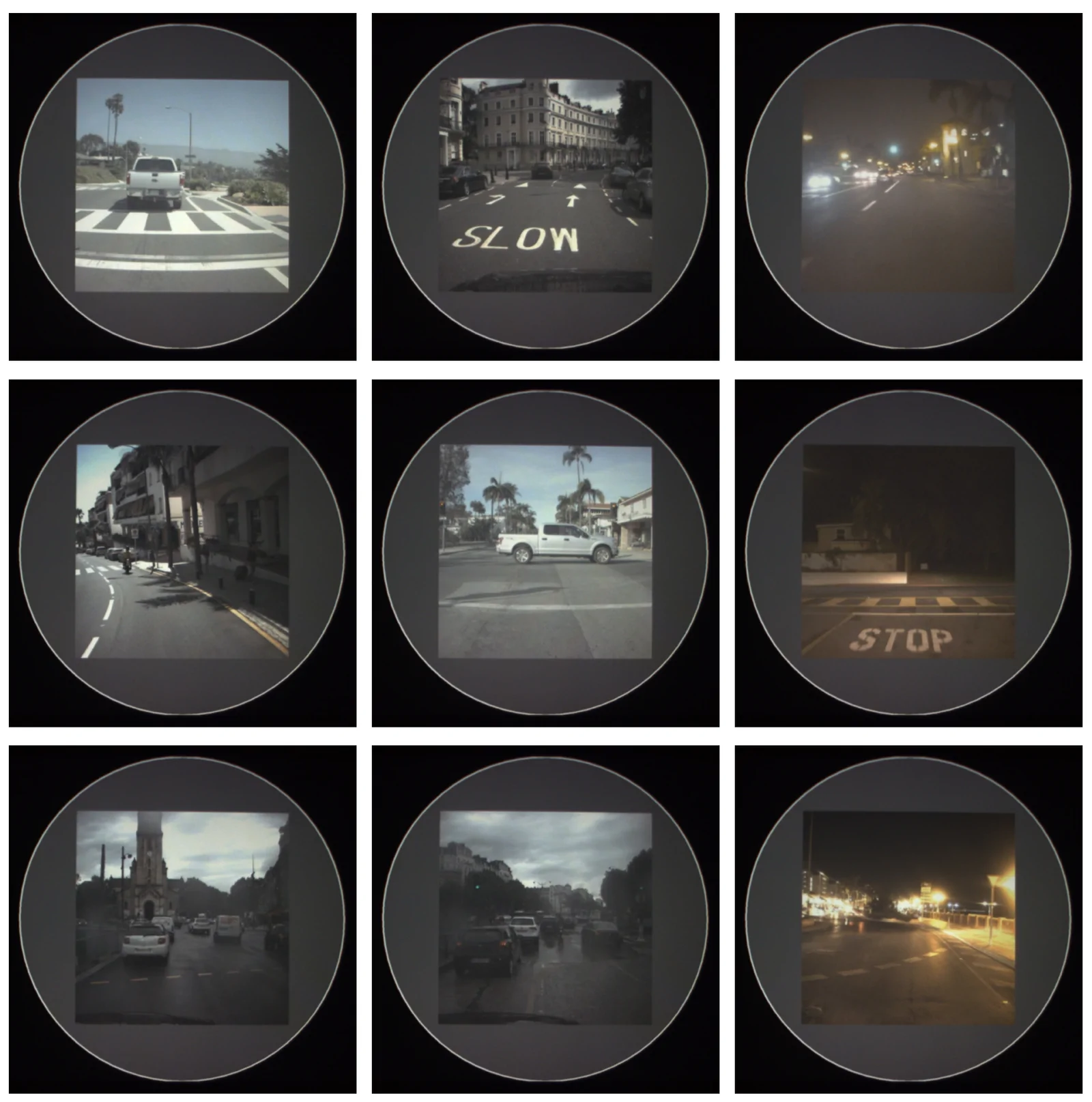
Examples of ground-truth images in our dataset. Original images were obtained from [12].

We first constructed a dataset consisting of paired scrambled images and their corresponding ground-truth images from road environments using the visible (RGB) frames of the Teledyne Forward-looking Infrared (FLIR) Advanced Driver Assistance Systems (ADAS) dataset [12]. To the best of our knowledge, there is no existing multicore, multimode fiber imaging dataset targeting road environments. The FLIR ADAS dataset contains road-environment data acquired in Europe and the United States, and the subset used in this study consisted of 11,777 image samples. Figure 7 presents examples of ground-truth images from the constructed dataset. The dataset includes both daytime and night-time data from typical road environments. For each of the ground-truth images, we created a corresponding scrambled image. For our experimental conditions, we set the lengths of the multicore, multimode optical fibers to 1.0, 5.0, and 10.0 m. We also constructed datasets for fibers with different core counts, including single-core, 217-core, 613-core, 1300-core, and 7400-core configurations. Figure 4 presents the image data for each core count configuration.

We captured the scrambled images using an optical bench, as illustrated in Figure 3. An LCD monitor presents each ground-truth image and serves as the target scene. An objective lens collects the displayed image and transfers it into the multicore, multimode optical fiber, which is a POF (the term “POF” in Figure 3 refers to this same fiber), and a second objective lens and an achromatic doublet relay the fiber output onto the image sensor of a camera module. Table 2 summarizes the specifications of the POFs used in this study. All configurations are step-index fibers and share a fiber diameter (core and cladding) of 1.0 mm and a numerical aperture (NA) of 0.5, whereas the coated outer diameter (including the jacket) is 1.25 mm for the 7400-core fiber and 2.20 mm for the others. This monitor-based configuration supports a controlled and repeatable approach to obtaining numerous precisely paired input and ground-truth images, which is essential for supervised training and isolating the effects of the fiber parameters (core count, fiber length, and calibration). Accordingly, the experiments described in this study aim to characterize the basic imaging performance of M3-RGB under controlled conditions. Section 5 systematically discusses the limitations of this controlled, display-based setup.

We randomly partitioned the image pairs into training, validation, and testing sets at an 8:1:1 ratio. We used the training set solely for model optimization, the validation set for model selection (i.e., for choosing the best-performing checkpoint during training), and the held-out testing set exclusively for final evaluations. To ensure fair comparisons across all fiber configurations, we applied identical partitioning to every core count and fiber length condition, meaning a given scene always belonged to the same subset. We generated the split using a fixed random seed to guarantee reproducibility. Unless otherwise stated, all reported quantitative results were computed based on the testing set.

For each experimental condition (i.e., each combination of core count, fiber length, and calibration setting), we trained an independent reconstruction model using the same architecture and hyperparameters. Accordingly, we evaluated condition-specific reconstruction performance, rather than generalizing a single model across all configurations. For reproducibility, Table 3 summarizes the training hyperparameters, which were identical for every fiber configuration.

Some of the experimental conditions involved rotation calibration of the scrambled images. In this study, we performed rotation calibration manually using a coarse-to-fine procedure; for each fiber configuration, we coarsely rotated the captured scrambled pattern into an approximately front-aligned orientation and then refined the angle in 1° steps, visually selecting the angle at which the rectangular outline of the transmitted road-scene image best matched the front-aligned orientation. This assessment relied on the rectangular outline, which remains discernible in the scrambled pattern owing to the spatial locality preserved by the multicore structure, not on the dense, nearly symmetric core lattice itself; the 180° ambiguity of the outline was resolved by the coarse scene layout (e.g., sky above, road below). The selected angles are thus discrete values on a 1° grid rather than sub-degree measurements, and we applied the same angle uniformly to all scrambled images of each configuration. Because we obtained this angle solely based on the appearance of the scrambled data and shared it across the training, validation, and testing splits, this calibration did not use the testing set ground-truth images. We applied calibration to the 217-core, 613-core, and 1300-core fibers. Regarding the 7400-core fiber, the captured scrambled pattern had already approximately matched the ground-truth orientation at the time of data acquisition (estimated rotation angle of approximately 0°); therefore, we did not apply rotation calibration. The single-core case was excluded because the rectangular outline was not discernible in single-core scrambled data. Section 4.4 discusses the effect of this calibration on reconstruction performance and the associated residual rotation error.

### 4.2. Effects of Core Count on Reconstruction Performance

**Figure 8:**
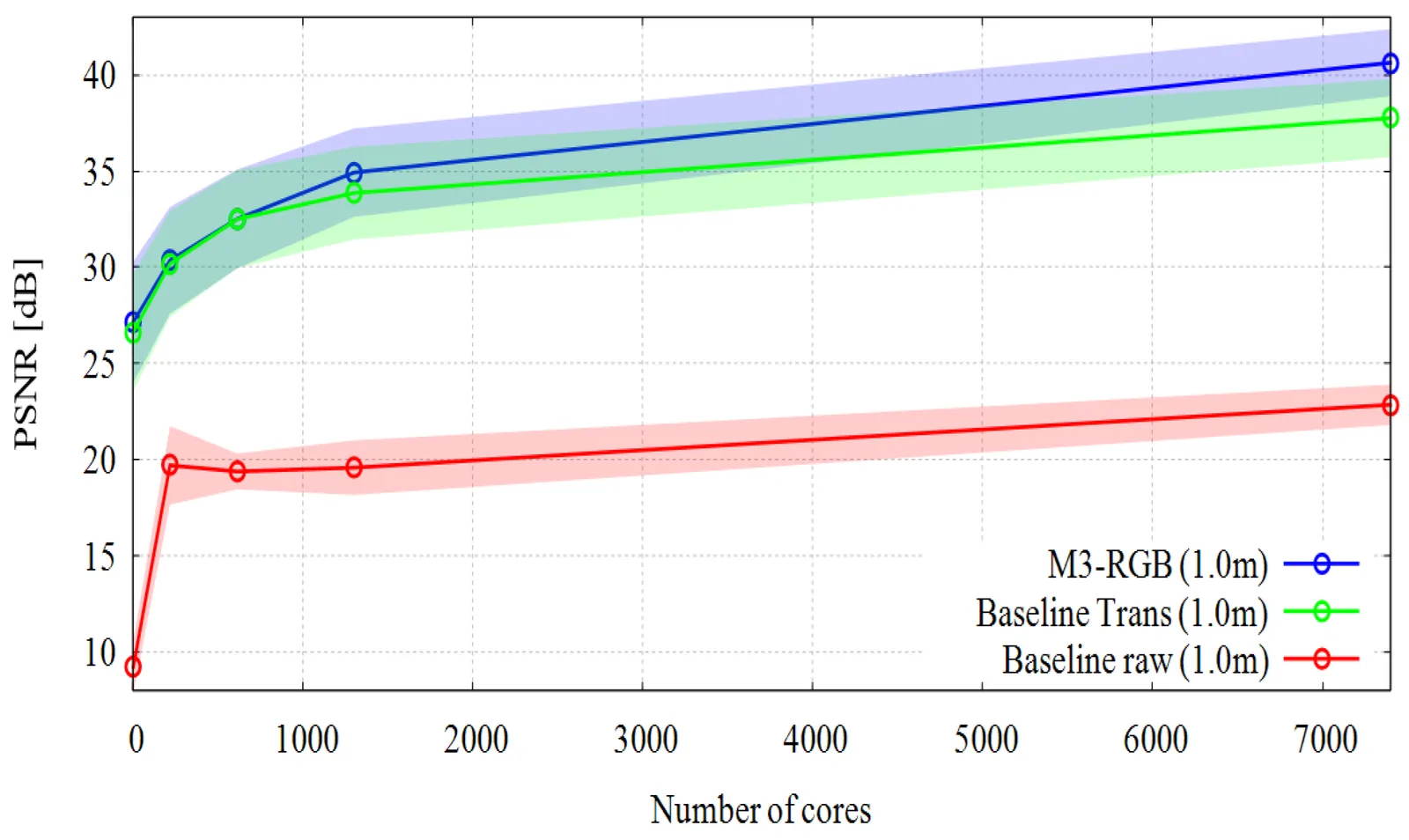
Peak signal-to-noise ratio (PSNR) evaluation across different numbers of cores using a 1.0 m multicore, multimode fiber. We obtained all the results without rotation calibration. This figure compares M3-RGB with the single-branch transformer baseline (Baseline Trans) and raw-input baseline (Baseline raw). Curves represent the mean outcomes over the testing set, and the shaded regions indicate ±1 standard deviation outcomes.

**Figure 9:**
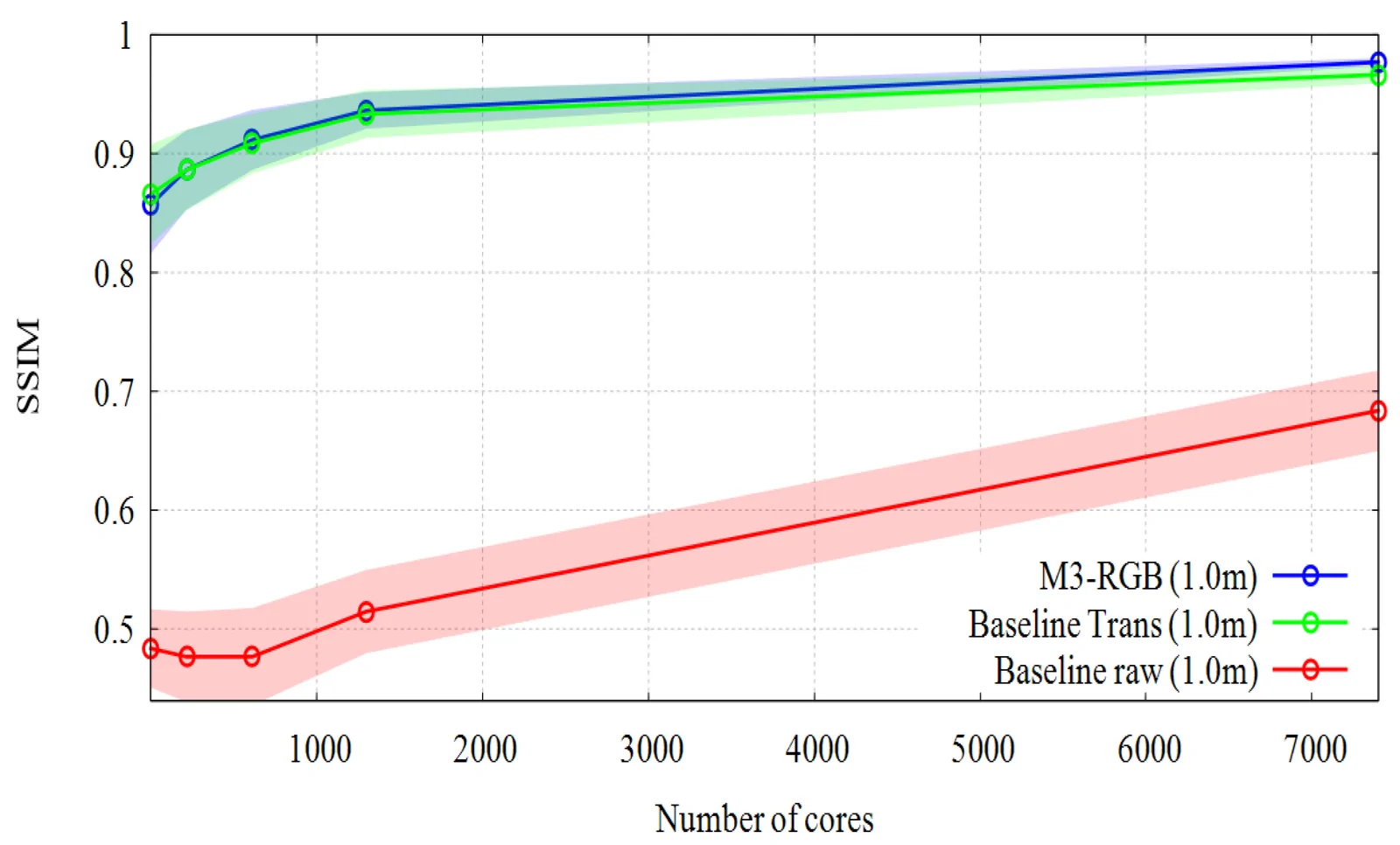
Structural similarity index measure (SSIM) evaluation across different numbers of cores using a 1.0 m multicore, multimode fiber. We obtained all results without rotation calibration. The figure compares M3-RGB with the single-branch transformer baseline (Baseline Trans) and raw-input baseline (Baseline raw). Curves represent the mean outcomes over the testing set, and the shaded regions indicate ±1 standard deviation outcomes.

**Figure 10:**
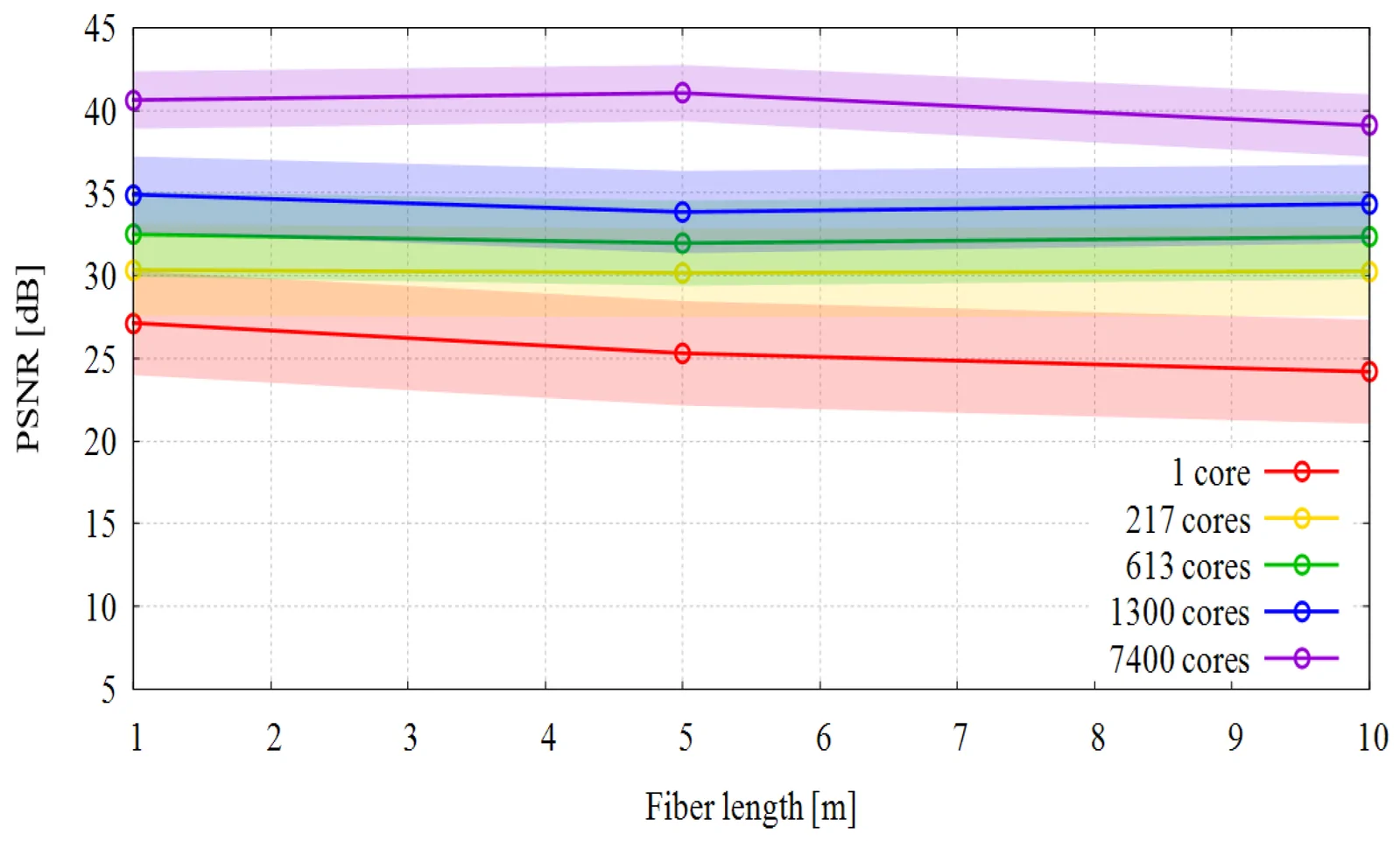
PSNR evaluation across different fiber lengths (1.0, 5.0, and 10.0 m) of the multicore, multimode fiber. Curves represent the mean outcomes over the testing set, and the shaded regions indicate ±1 standard deviation outcomes.

**Figure 11:**
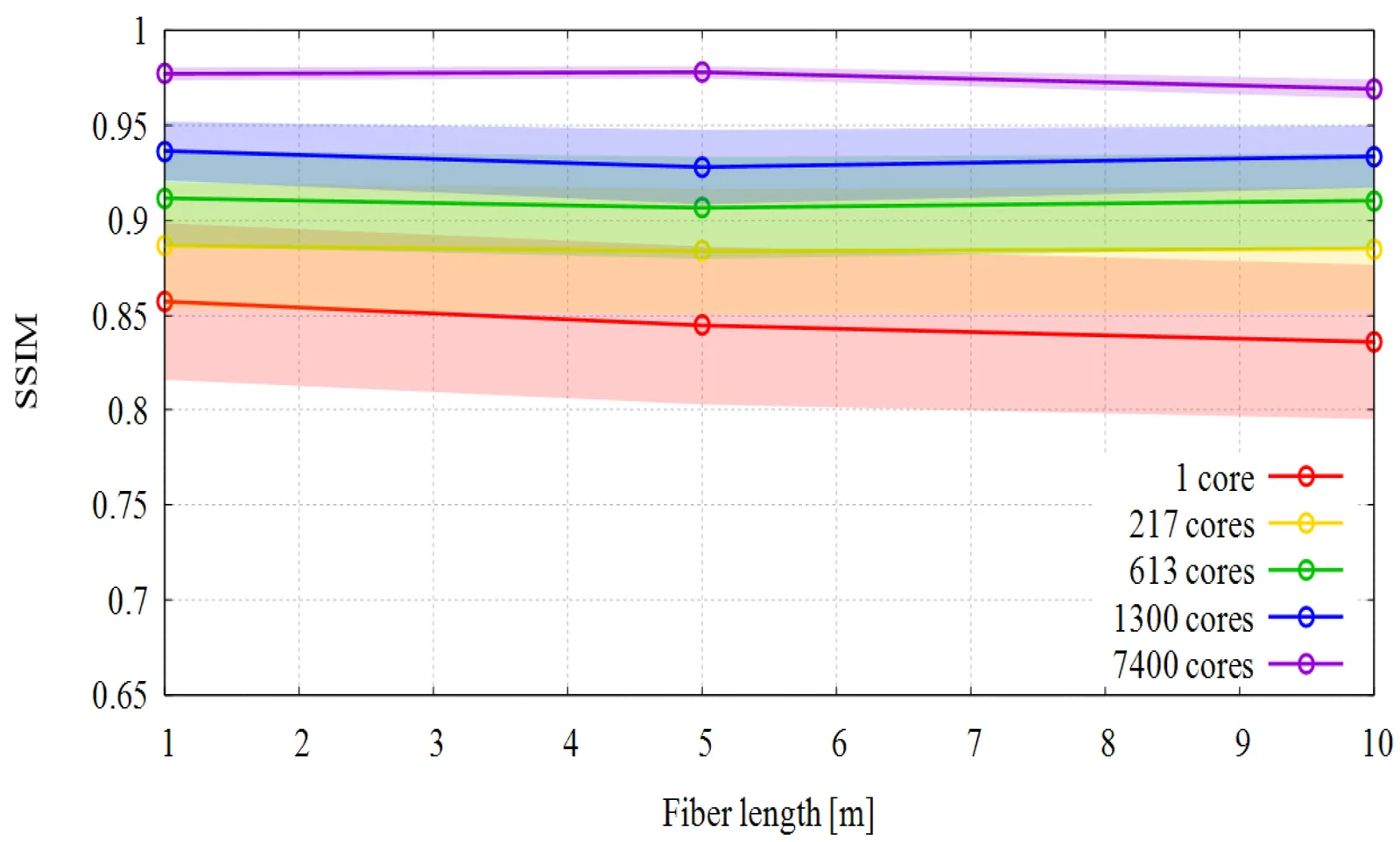
SSIM evaluation across different fiber lengths (1.0, 5.0, and 10.0 m) of the multicore, multimode fiber. Curves represent the mean outcomes over the testing set, and the shaded regions indicate ±1 standard deviation outcomes.

In our first experiment, we evaluated the effects of varying the number of cores in the multicore fibers on image reconstruction performance. By using the aforementioned dataset, we conducted image reconstruction experiments and quantitatively assessed reconstruction performance. The evaluation metrics were the peak signal-to-noise ratio (PSNR) and structural similarity index measure (SSIM). We report all the quantitative results as the mean values over the testing set. The shaded regions in Figure 8, Figure 9, Figure 10 and Figure 11 indicate ±1 sample standard deviation across the testing images, and Table 4 likewise reports the mean ± one standard deviation. We compared M3-RGB against two baselines: the unprocessed scrambled image itself and a vanilla single-branch transformer. First, as a reference, we report a *raw input baseline*, where we compute the PSNR and SSIM directly between the scrambled input image (i.e., the raw image transmitted through the fiber, resized to the evaluation resolution) and the corresponding ground-truth image, without applying any reconstruction. This baseline corresponds to replacing the network output with the raw fiber-transmitted input, effectively quantifying the intrinsic difficulty of each fiber configuration and isolating the contribution of the reconstruction network. Second, to assess the contribution of the parallel two-branch design of M3-RGB, we report the results of a *transformer baseline* (denoted as “Baseline Trans” in the figures), where we replace the reconstruction network with a vanilla single-branch ViT using a single patch size and single positional embedding, without the parallel branches or attention-based fusion block of M3-RGB. We trained this baseline on the same dataset with the same data splits and training hyperparameters, using a standard (unweighted) L1 reconstruction loss. Figure 8 and Figure 9 present the results of the first experiment with the two baselines. We obtained all the results in this experiment without rotation calibration, and the effects of calibration were evaluated separately in Section 4.4. As shown, both the PSNR and SSIM increase as the number of cores increases from the single-core configuration. This trend is attributable to the fact that the scrambling and associated loss of spatial information become increasingly localized as the number of cores increases. Section 5.1 discusses the underlying mechanism in more detail. The results reveal that M3-RGB matches or outperforms the single-branch transformer baseline in terms of PSNR across all core counts, and the margin broadens in the high-core-count regime, reaching 2.88 dB in the 7400-core configuration (Figure 8). In terms of SSIM, the difference between the two models is marginal (Figure 9), indicating that the benefit of the parallel two-branch design manifests primarily as a reduction in pixel-wise reconstruction error, rather than in structural similarity.

### 4.3. Effects of Fiber Length on Reconstruction Performance

**Figure 12:**
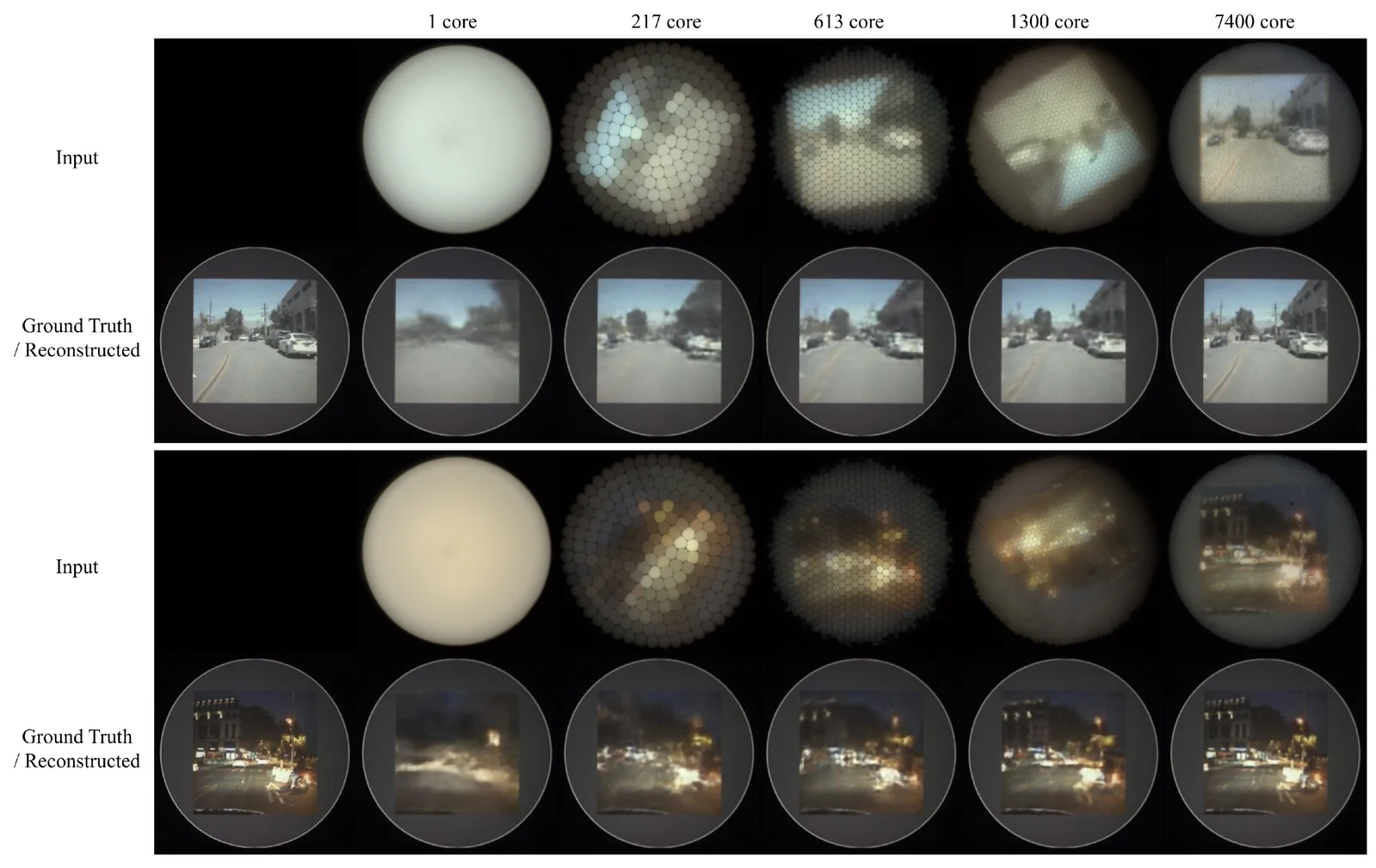
Examples of input, reconstructed, and ground-truth images for different core counts. The upper block represents a daytime scene, while the lower block represents a night-time scene. In each block, the top row shows the scrambled input for each core count, and in the bottom row, the leftmost image is the ground truth, while the remaining columns show the corresponding reconstructions for all core counts.

In our second experiment, we evaluated the performance differences resulting from changes in the fiber length. The first experiment used a 1.0 m fiber. In this experiment, we extended the fiber length to 5.0 and 10.0 m. Figure 10 and Figure 11 depict plots of the PSNR and SSIM as functions of the fiber length. It can be observed that even as the fiber length increases to 5.0 and 10.0 m, image reconstruction performance consistently improves as the number of cores increases. Additionally, Figure 12 presents examples of the input scrambled images and corresponding reconstructed images for each core count configuration.

Interestingly, the 10.0 m fiber yields slightly higher PSNR and SSIM outcomes than those obtained by the 5.0 m fiber for the 217-, 613-, and 1300-core configurations. Because attenuation, intermodal dispersion, and mode mixing can only degrade the signal with increasing length, we attribute this to session-to-session differences in acquisition: the fiber section (input coupling and output imaging optics) was reassembled for each length, while the camera exposure and gain remained constant. Indeed, the 10.0 m inputs are consistently brighter (e.g., a mean intensity of 61.7 for the 613-core configuration versus 50.1 at 1.0 m and 38.8 at 5.0 m), sharper, and less vignetted than the 5.0 m inputs—differences a longer fiber cannot produce—indicating that coupling and focusing quality dominate the length conditions. This is plausible over the range of 1.0–10.0 m, where the accumulated attenuation is modest, intermodal dispersion is integrated within the camera exposure, and the intercore spatial segmentation providing the dominant reconstruction cue is preserved regardless of length. Figure 10 and Figure 11 show that reconstruction quality is maintained for length values at least up to 10.0 m, with the core count trend maintained at every length, rather than expressing a monotonic dependence on length itself.

### 4.4. Effects of Calibration on Reconstruction Performance

In our third experiment, we evaluated the performance differences resulting from calibration. Specifically, we evaluated image reconstruction performance when the scrambled data shown in Figure 4 were used directly and when images were reconstructed after calibrating the rotation of the scrambled data following the manual calibration procedure described in Section 4.1. Table 4 presents representative quantitative results for the 1.0 m fiber, with and without calibration, wherever applicable. All the results presented in Figure 8 and Figure 9 were obtained without calibration. The evaluation results reveal that calibration provides a consistent, albeit modest, performance improvement.

We also analyzed the residual error of this calibration. Because the angles were selected on a 1° grid, the residual rotation error is within ±0.5°. A 1° rotation corresponds to a displacement of at most approximately 9μm at the fiber periphery (radius 0.5 mm; Table 2), smaller than the core pitch (approximately 26μm for the 1300-core fiber); the residual misalignment therefore remains sub-core. Moreover, it acts as a systematic offset shared by all the data splits of each configuration and is absorbed by the condition-specific training.

## 5. Discussion and Limitations

The experiments reported earlier established the basic imaging performance of M3-RGB under controlled optical bench conditions. In this section, we clarify the nature of the target reconstruction problem in relation to general image restoration and super-resolution and discuss the underlying reconstruction mechanism and then summarize the limitations of this study systematically, in conjunction with the practical implications of the passive sensing architecture.

### 5.1. Relationship with Image Restoration and Super-Resolution

As shown in Section 3, increasing the number of cores makes the transmitted scene progressively more recognizable because the scrambling and associated spatial information loss become localized in the regions of individual cores. A natural question is whether the task solved here is genuinely a fiber channel inversion or actually a form of low-resolution or rotated image restoration or super-resolution. The proposed reconstruction problem is not a purely blind inversion of fully randomized speckle patterns. In high-core-count configurations, the multicore structure preserves partial spatial correspondence between the scene and captured pattern, meaning the target task lies between fiber channel inversion and image restoration/super-resolution. This partial preservation of spatial locality is not regarded as a confounding factor but as a design feature of the proposed sensing architecture. By employing a multicore, multimode fiber, M3-RGB deliberately trades the fully scrambled, single-core speckle regime for a regime retaining spatial locality, which makes reconstruction tractable. In the single-core limit, where this type of locality is unavailable, the problem is reduced to a genuinely blind speckle inversion, and reconstruction quality degrades accordingly, consistent with the core count dependence observed in our experiments.

Specifically, the multicore fiber acts as a spatial sampler of the relayed image: light is scrambled within each core by intermodal dispersion and mode coupling, whereas the core-to-core arrangement is fixed by the fiber geometry and preserves the spatial ordering of the scene. The core count thus sets the effective sampling density—for the 1.0 mm fiber diameter (Table 2), the hexagonal core pitch decreases from approximately 64 μm (217 cores) to 11 μm (7400 cores)—and reconstruction reduces to a locally conditioned restoration problem of inverting intracore smearing and the honeycomb sampling pattern, akin to joint deblurring and demosaicking. With fewer cores, more information must be recovered from the strongly mixed within-core distributions, degenerating into a global blind speckle inversion in the single-core limit, which explains the monotonic improvement in PSNR and SSIM with the core count observed in Section 4.

Regarding the number of guided modes, a single well-defined mode number cannot be assigned to each configuration because the individual core diameters of the multicore POFs are not provided in the manufacturer specifications and the system operates on broadband incoherent light, rather than at a single wavelength. Nevertheless, a scaling argument holds: all configurations share approximately the same total core cross-section and NA (Table 2); therefore, the total number of modes supported by the fiber is approximately invariant across core counts, whereas the number of modes per core decreases as the individual cores become smaller with increasing core count. The monotonic improvement with the core count indicates that reconstruction performance is governed by how this (approximately) constant mode resource is spatially partitioned into cores—that is, by the multicore architecture and its spatial locality rather than by the total number of guided modes. A rigorous characterization of mode-number dependence would require a dedicated set of fibers in which the structural and propagation parameters are independently controlled. This analysis constitutes future work.

### 5.2. Limitations

The following limitations should be considered when interpreting the results presented above.

*Display-based target and controlled conditions.* Because the targets in this study were images displayed on an LCD monitor rather than a real outdoor 3D scene, the properties of the display, including luminance, spectral characteristics, gamma, refresh rate, polarization, and viewing angle, remained fixed. Consequently, our setup did not reproduce effects specific to natural scenes, including scene depth, object motion, actual nighttime and low-light illumination levels (as opposed to displayed night-time imagery), backlighting, and high-dynamic-range conditions. Therefore, the reported performance measures characterize the fiber-based imaging channel under controlled conditions and do not represent an evaluation in real-world driving environments.

*Condition-specific reconstruction models.* As noted in Section 4, we trained an independent reconstruction model for each experimental condition (i.e., each combination of core count, fiber length, and calibration setting), utilizing the same architecture and hyperparameters. Therefore, our evaluations targeted condition-specific reconstruction, rather than a single model generalized across configurations. Accordingly, we did not assess the generalization of a particular model to unseen fiber lengths, core counts, or calibration states.

*Scope of the sensing configuration.* Our evaluations used a single image sensor to capture a single fiber end face, and we quantified reconstruction quality based on the PSNR and SSIM values on the held-out testing set. Multi-end-face acquisition, task-level perception metrics, and robustness to fiber bending, coupling misalignment, and temperature variation are outside the scope of this work. Nevertheless, several properties of the proposed configuration suggest a degree of inherent robustness. First, unlike coherent transmission-matrix-based multimode fiber imaging, whose speckle patterns decorrelate under bending and temperature drift conditions, M3-RGB operates solely on incoherent intensity patterns and is therefore intrinsically less sensitive to phase perturbations. Second, the dominant reconstruction cue—the intercore spatial segmentation—is anchored to the physical core lattice of the fiber, and mechanical or thermal perturbations mainly redistribute intensity within individual cores rather than altering the intercore mapping. Third, Fukushima et al. [4] have empirically demonstrated robustness to bending, twisting, and temperature variation for incoherent-light transmission through a single-core POF. Conversely, as observed in the fiber-length experiments (Section 4), absolute performance depends on the coupling and focusing quality of the assembled optics, and recalibration or retraining would be required after a major reconfiguration. We consider that the experimental establishments of robustness for the multicore configuration constitute future work.

*Manual rotation calibration.* As described in Section 4, we performed rotation calibration manually, selecting a single global rotation angle for each fiber configuration on a 1° search grid by visually aligning the rectangular outline of the transmitted image. This manual procedure cannot be easily applied to the single-core case where the rectangular outline is not discernible, and it does not scale reasonably to a large number of configurations. We consider that automating the estimation of the rotation angle, which will allow calibration to proceed without visual inspection and extend to the single-core regime, constitutes future work. In particular, we plan to replace the current visual inspection method with an automatic estimation scheme, potentially based on a checkerboard or fiducial-marker target or on phase-correlation alignment between the captured and reference patterns.

*Session-dependent acquisition conditions.* The datasets for different fiber lengths were acquired in separate sessions in which the fiber section was reassembled. Therefore, differences in coupling and focusing quality are confounded with the fiber length (Section 4); comparisons across core counts within each length condition are unaffected. A strictly controlled length comparison with an identical coupling setup constitutes future work.

*Optical throughput and coupling efficiency.* The optical throughput of the fiber relay is inherently lower than that of a direct free-space camera owing to the aperture and NA mismatch at the input coupling stage, intercore cladding regions of the multicore fiber (fill-factor loss), propagation attenuation, and the output relay optics. Because these losses depend strongly on the design of the coupling optics, measurements with the present bench-mounted objective lenses would characterize only the current proof-of-concept setup rather than a future miniaturized implementation. The quantitative evaluation of the coupling efficiency, total throughput, and the resulting SNR penalty under actual low-light conditions constitutes future work in conjunction with the compact coupling optics described in Section 6.

These limitations do not affect the findings reported for controlled conditions, but they specify the conditions under which the results hold and directly motivate the directions outlined below.

Regarding practical applicability, the passive nature of the sensing interface is associated with several implications beyond the placement flexibility discussed above: the sensing head requires no electrical power and dissipates no heat, the optical fiber link is immune to electromagnetic interference, and the absence of electrical components at the distal end is advantageous in adverse or explosion-proof environments, such as the pipeline inspection scenario in Section 6. In addition, POFs are inexpensive, lightweight, and easy to handle compared with glass fibers, and the core count/cost trade-off discussed in Section 6 provides a practical design parameter for matching the system to application requirements.

## 6. Future Work

**Figure 13:**
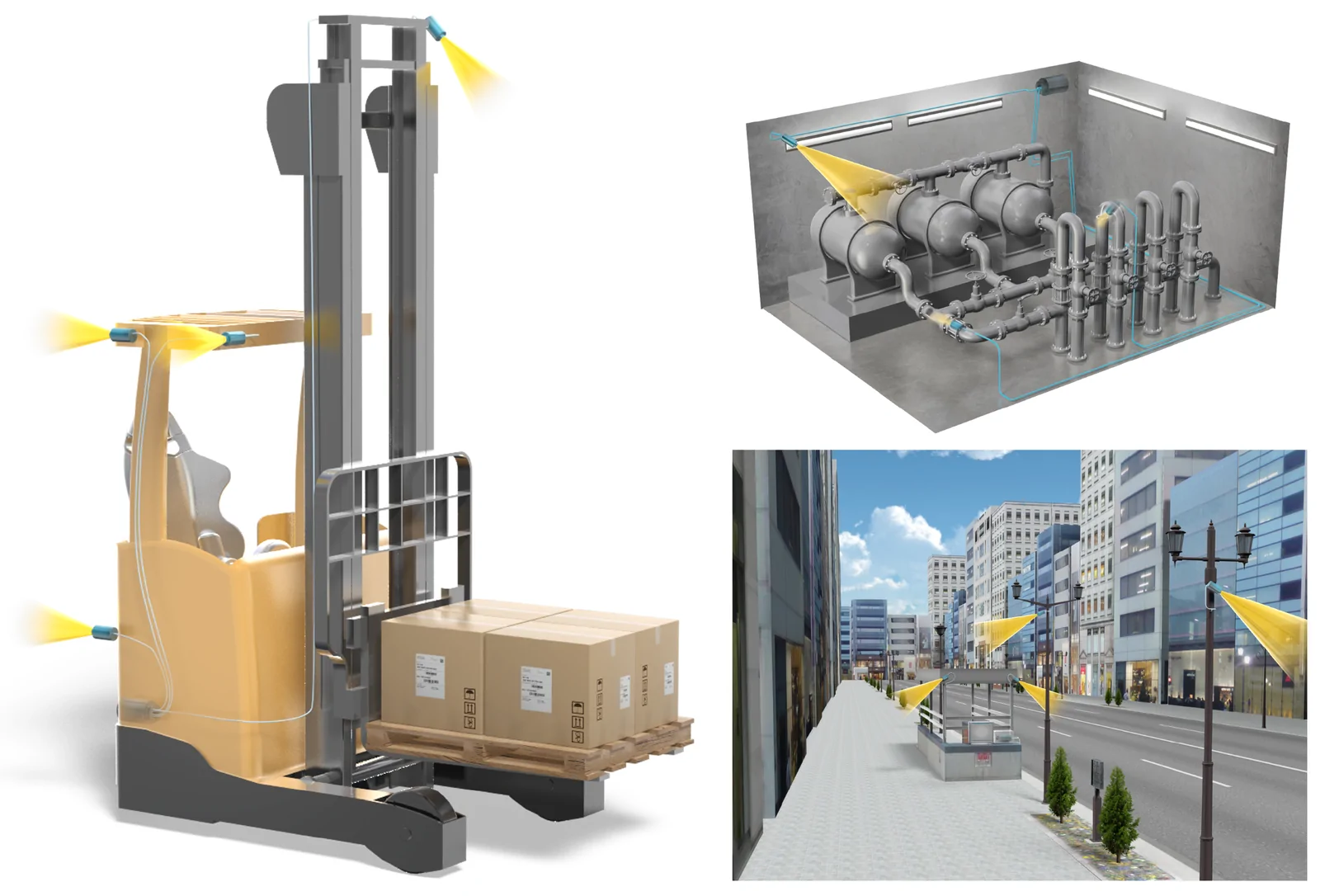
Illustrative applications envisioned for M3-RGB, including peripheral sensing for autonomous forklifts, substitution for conventional cameras in urban surveillance systems, and inspection tasks within narrow environments, such as pipelines. These scenarios represent envisioned directions; accordingly, we did not evaluate them experimentally in this study.

We envision several application scenarios for M3-RGB (see Figure 13). First, M3-RGB can support all-around visual sensing for mobile robots by enabling flexible sensor placement and reducing the number of image sensors required on platforms. Second, the proposed system is well-suited to replacing conventional surveillance cameras, as its passive fiber-based image relay allows imaging electronics to reside remotely from the sensing interface. Third, M3-RGB can support inspection tasks in confined or hard-to-access environments, such as pipelines or narrow industrial spaces where compact sensing interfaces and remote image transmission are advantageous. We envision these application scenarios as promising future developments but did not evaluate them experimentally in this study.

Regarding fiber selection in these scenarios, the experiments described in this study clarified the relationship between the number of fiber cores and reconstruction performance. However, fiber cost increases with the core count. Therefore, in future deployments, we plan to select an appropriate fiber type for each application by weighing the cost that an application allows against the reconstruction performance it requires.

Realizing these scenarios requires closing the gap between the present controlled bench characterization and real-world operation. The experiments in this study validate image reconstruction through the fiber channel itself, using pre-acquired road-scene images displayed on an LCD monitor and bench-mounted objective lenses. The subsequent steps toward the envisioned applications include the (i) replacement of the bench objective lenses with compact lenses integrated into the sensing head, allowing the system to be deployed in more diverse environments, and (ii) the shifting from the pre-acquired, display-based dataset to the direct acquisition of real-environment scene images with the proposed system and the evaluation of their reconstruction, including outdoor dynamic scenes and cross-configuration generalization. In addition, in this study, we used a single image sensor to capture only one fiber end face. In the next step, we will extend M3-RGB to a system in which a single image sensor can simultaneously capture multiple fiber end faces, enabling the comprehensive acquisition of the surrounding environment in a single shot. In this context, recent advances in metalenses and metalens arrays are promising routes toward miniaturized, lightweight sensing heads: ultrathin meta-lenses with advanced wavefront control [13] could serve as compact alternatives to the objective and coupling lenses at the passive sensing interface, and varifocal metalens arrays demonstrated for adaptive light-field imaging [14] are well-suited to coupling multiple fiber end faces onto a single image sensor in the envisioned multifiber configuration.

## 7. Conclusions

In this study, we introduced M3-RGB as an incoherent-light fiber imaging system that passively relays lens images to a remotely located image sensor through a multicore, multimode optical fiber and reconstructs captured scenes using a neural reconstruction network and rotation calibration. Under controlled optical bench conditions, where road-scene images displayed on an LCD monitor served as the targets, we constructed a paired dataset of scrambled and ground-truth images and quantitatively characterized reconstruction performance across different fiber core counts, fiber lengths, and calibration settings. Our findings remain limited to this controlled optical bench characterization. Validation on real outdoor scenes constitutes future work. Ultimately, we aim to advance the development of next-generation camera systems that are capable of high-fidelity imaging through complex optical channels.

## Figures and Tables

**Table 1:** Positioning of M3-RGB relative to representative fiber-based imaging studies. “Incoherent scene light” indicates operation on incoherent light from a displayed or real scene (demonstrated here with scenes displayed on liquid crystal displays), without a laser source, an SLM, or a dedicated illumination unit at the fiber sensing interface. “Demonstrated distance” denotes the fiber lengths experimentally demonstrated in each study (—: not reported).

Method	Light Source	Fiber Type	Demonstrated Distance	Target Content
Caramazza et al. [3]	Laser + SLM	Single-core, multimode	1 m, 10 m	Natural scenes (ImageNet)
Liu et al. [8]	Pulsed laser	Single-core, multimode	1 km	Handwritten digits, letters
Zheng et al. [9]	Active multiwavelength probing	Multimode	1–2 m	Transmission-matrix recovery
Hu et al. [10]	Dedicated illumination	Disordered fiber	∼0.8 m	Cellular specimens
Shao et al. [11]	Dedicated illumination	Fiber bundle	—	Microscopy targets
Fukushima et al. [4]	Incoherent scene light	Single-core plastic	0.1–10 m	Handwritten characters, digits
**M3-RGB (ours)**	**Incoherent scene light**	**Multicore, multimode plastic**	**1.0–10.0 m**	**Road-scene natural images**

**Table 2:** Specifications of the multicore, multimode plastic optical fibers used in the experiments. The fiber diameter denotes the diameter of the core and cladding region, and the coated outer diameter includes the jacket.

Cores	Coated Outer Diameter [mm]	Fiber Diameter [mm]	Numerical Aperture
1	2.20	1.0	0.5
217	2.20	1.0	0.5
613	2.20	1.0	0.5
1300	2.20	1.0	0.5
7400	1.25	1.0	0.5

**Table 3:** Training hyperparameters of M3-RGB. We used identical settings for all fiber configurations. Only the fiber parameters (core count, fiber length, and calibration state) differed across experiments.

Parameter	Value
Optimizer	Adam
Learning rate	$1\times 10^{-4}$
Batch size	20
Epochs	200
Loss	Channel-weighted reconstruction loss
Channel weights s (R, G, B)	(1.8,1.3,1.4)

**Table 4:** Representative image reconstruction performance of M3-RGB with the 1.0 m fiber across different core counts and calibration settings. Mean ± standard deviation over the testing dataset. ΔPSNR denotes the improvement obtained by calibration relative to the corresponding uncalibrated configuration. The calibrated angle denotes the global rotation applied to the scrambled images, selected manually on a 1° grid by aligning the rectangular outline of the transmitted image (positive angles are counterclockwise; Section 4.1); −210° is equivalent to +150°. Figure 8 and Figure 9 illustrate the corresponding core count trends without calibration, while this table summarizes representative quantitative results with and without calibration where applicable. We do not apply calibration to the single-core case, and we omit the calibrated row for the 7400-core fiber because its captured pattern already approximately matched the ground-truth orientation at the time of data acquisition (Section 4.1).

Cores	Calibration	Calibrated Angle [deg]	Peak Signal-to-Noise Ratio (PSNR) ↑ [dB]	ΔPSNR [dB]
1	–	–	27.15 ± 3.15	–
217	–	–	30.36 ± 2.77	–
217	✓	−62	30.50 ± 2.76	+0.14
613	–	–	32.51 ± 2.37	–
613	✓	−10	32.83 ± 2.59	+0.32
1300	–	–	34.92 ± 2.30	–
1300	✓	−210	35.22 ± 2.36	+0.30
7400	–	–	40.63 ± 1.74	–

## Data Availability

Requests for access to the data presented in this study will be considered by the corresponding author on a case-by-case basis. The ground-truth images used in this study were obtained from the third-party FLIR ADAS Dataset [12], which is available from FLIR under its own license terms; the paired fiber-transmission images are derived from these images and therefore cannot be publicly redistributed by the authors.
